# Multifunctional Oregano‐Derived Plasma Polymer Coatings for Wound Healing Applications: An In Vitro Study

**DOI:** 10.1111/iwj.70977

**Published:** 2026-06-17

**Authors:** Jesus Romo‐Rico, Neethu Ninan, Andrew Hayles, Smriti Murali Krishna, Krasimir Vasilev, Jonathan Golledge, Richard Bright, Mohan Jacob

**Affiliations:** ^1^ Electronics Materials Lab, College of Science and Engineering James Cook University Townsville Queensland Australia; ^2^ Queensland Research Centre for Peripheral Vascular Disease James Cook University Townsville Queensland Australia; ^3^ College of Medicine and Public Health Flinders University Bedford Park South Australia Australia; ^4^ Atherothrombosis and Vascular Biology Baker Heart and Diabetes Institute Melbourne Victoria Australia; ^5^ Department of Vascular and Endovascular Surgery Townsville University Hospital Townsville Queensland Australia

**Keywords:** antibacterial, anti‐inflammatory, oregano, plasma‐polymerisation, wound healing

## Abstract

Chronic wounds, exacerbated by infection, aging, diabetes, and oxidative stress, remain a significant challenge in clinical management and impose a substantial burden on healthcare systems. This study presents oregano‐derived plasma polymers (OPP) as a multifunctional surface coating for advanced wound healing applications. OPP coatings were fabricated using radiofrequency plasma‐enhanced chemical vapour deposition and deposited on glass coverslips. Surface chemistry was characterised by x‐ray photoelectron spectroscopy and Fourier‐transform infrared spectroscopy, and hydrophilicity was assessed by water contact angle measurements. Antibacterial activity against *Pseudomonas aeruginosa* and *Staphylococcus aureus* was evaluated using LIVE/DEAD staining and confocal microscopy. Cytocompatibility, cell adhesion, and morphology were analysed using RAW 264.7 macrophage‐like cells, as assessed by MTT assays and immunofluorescent staining for actin and nuclei. The immune response was evaluated by quantifying the cytokines, IL‐4 and IL‐6 using an enzyme‐linked immunosorbent assay. Antioxidant properties were assessed by measuring lipopolysaccharide‐induced intracellular reactive oxygen species via fluorescence imaging. The pro‐regenerative potential was evaluated using in vitro scratch assays with human foreskin fibroblasts, monitored over 30 h with live‐cell imaging. OPP surfaces retained key bioactive chemical groups and exhibited enhanced hydrophilicity. They significantly reduced bacterial viability ( *P. aeruginosa* : ~90%, *S. aureus* : ~40%, *p* < 0.01) and supported macrophage adhesion, spreading, and viability. OPP downregulated IL‐4 expression without elevating IL‐6, suggesting a favourable immunomodulatory profile. OPP coatings inhibited LPS‐induced reactive oxygen species production (*p* < 0.0001), demonstrating antioxidant activity. Scratch assays confirmed significantly accelerated fibroblast migration and wound closure on OPP surfaces (*p* < 0.05). OPP coatings combine antibacterial, anti‐inflammatory, antioxidant, and pro‐regenerative properties with excellent cytocompatibility, offering a scalable plant‐based platform for advanced wound healing and next‐generation nature‐inspired biomaterials. This study is the first to demonstrate that oregano‐derived plasma polymer coatings possess combined antibacterial, antioxidant, immunomodulatory, and pro‐regenerative properties relevant to wound healing applications. Future work will focus on in vivo validation and manufacturability optimisation for clinical translation.

## Highlights

- Oregano‐derived plasma polymer coatings significantly reduce bacterial viability while supporting macrophage viability and adhesion.
- The coatings suppress oxidative stress, modulate inflammatory responses, and accelerate fibroblast‐mediated wound closure in vitro.
- OPP coatings represent a multifunctional, plant‐based biomaterial platform with potential applications in advanced wound dressings and regenerative medicine.

## 1 Introduction

The growing prevalence of systemic diseases, an increasingly aging population, and trauma‐related injuries have contributed to a dramatic rise in chronic wound cases [1]. Diabetic ulcers and pressure sores, as examples of chronic wounds, are challenging due to their prolonged healing process and susceptibility to infections [2]. Wound dressings have been continually improved to address chronic wounds, yet effective wound management remains a significant challenge [3]. When a wound dressing comes into contact with tissue, the body's immune system recognises it as a foreign substance and initiates an acute inflammatory response [4]. If this inflammation becomes prolonged, it can result in a chronic wound, leading to persistent pain, limited mobility, diminished physical health, and a higher likelihood of infection, alongside substantial healthcare expenses, accompanied by a representative healthcare cost [5, 6]. As a result, there is a growing need for advanced wound dressing materials that promote the healing of chronic wounds, such as hydrogels derived from both synthetic and natural polymers. These materials are increasingly recognised for their tunable mechanical properties, biocompatibility, and ability to support tissue regeneration [7]. An ideal wound healing material should possess multiple beneficial properties. First, it should avoid triggering the host's immune response to prevent unnecessary inflammation [8], which can delay healing. Approaches that combine antimicrobial agents with collagen coatings have shown promise for producing wound‐contact materials with favourable wettability and antibiofilm activity [9].

Additionally, it should possess antioxidant capabilities to reduce oxidative stress [10], a critical factor in chronic inflammation, and recent multifunctional wound‐healing biomaterials have increasingly incorporated ROS‐scavenging strategies to improve tissue repair and inflammatory regulation [11, 12]. Biological validation of collagen‐coated PCL membranes functionalised with silver nanoparticles demonstrated robust fibroblast adhesion and preserved viability, highlighting the need to balance antimicrobial efficacy with cytocompatibility in wound dressing design [13]. Furthermore, the material should actively promote cell proliferation, allowing for healthy tissue regeneration and supporting tissue remodelling processes to restore the structural and functional integrity of the damaged area [14]. These combined features are crucial in addressing the complex nature of chronic wound healing. There has been extensive research on metal‐based technologies to address wound healing [15, 16, 17]. However, plant‐based alternatives may offer more environmentally friendly solutions.

In recent years, natural and plant‐based compounds have attracted significant attention as promising alternatives for wound‐healing applications. Many plant‐derived compounds, such as *aloe vera* , garlic, curcumin, and essential oils, have demonstrated excellent therapeutic properties, including anti‐inflammatory, antioxidant, and antimicrobial effects [18]. These natural compounds can be incorporated into wound dressings to enhance healing while reducing the risk of adverse reactions often associated with synthetic materials [18]. For instance, *aloe vera* is recognised for its soothing and moisturising properties [16], promoting faster epithelialisation and reducing inflammation. With its potent anti‐inflammatory and antioxidant characteristics, curcumin can mitigate oxidative stress and accelerate tissue regeneration [19]. Garlic, a widely studied natural agent, has been shown to enhance wound healing and possess notable antibacterial properties [20]. Moreover, natural compounds are biocompatible, eco‐friendly, and often more cost‐effective than synthetic alternatives, making them attractive for the development of next‐generation wound‐healing materials [21]. These plant‐based therapies offer a holistic approach to wound care and align with the growing trend toward sustainable and nature‐inspired biomedical solutions.

Oregano is an aromatic herb with a long‐standing tradition of being used for wound healing. Multiple studies have shown that oregano's antibacterial, antioxidant, and anti‐inflammatory properties significantly promote wound healing [22, 23, 24]. Ointments [25], hydrogels [26], nanofibers [27], nanoparticles [28] and films [29] are examples of engineered materials developed to incorporate oregano extracts for their wound‐healing properties. These materials utilise the bioactive compounds in oregano, such as carvacrol and thymol, which possess strong antimicrobial, anti‐inflammatory, and antioxidant effects that can enhance wound healing [30]. For instance, nanofibers made from collagen hydrolysate and infused with oregano essential oil (OEO) have demonstrated excellent in vitro biocompatibility with fibroblast cells, a key cell type involved in tissue repair [31]. These nanofiber scaffolds provide structural support that mimics the extracellular matrix, facilitating the controlled release of oregano‐derived bioactive compounds that can promote cell proliferation and reduce the risk of infection. Similarly, hydrogels incorporating oregano extracts have been explored for their ability to maintain a moist wound environment while delivering antimicrobial and anti‐inflammatory agents directly to the wound site [26]. Films and ointments infused with oregano essential oil have also shown potential to accelerate wound closure and reduce bacterial contamination. By integrating oregano extracts into these advanced wound‐healing materials, researchers aim to harness oregano's natural therapeutic properties while overcoming limitations, such as volatility and stability concerns, associated with direct application. These engineered materials represent a promising avenue for developing effective, biocompatible, and sustainable wound care solutions.

They demonstrated antioxidant and antibacterial properties, as well as the ability to eradicate biofilms [31]. OEO encapsulated in chitosan‐alginate nanoparticles showed no signs of skin irritation or swelling when tested on rabbits [32]. Another study encapsulated OEO within poly(L‐lactide‐co‐ε‐caprolactone) and silk fibroin nanofiber (NF‐OEO) membranes [33]. These NF‐OEO membranes significantly enhanced in vivo wound healing by stimulating angiogenesis, granulation tissue formation, neo‐epithelialisation, and collagen deposition. Moreover, the membranes exhibited antibacterial properties against Gram‐positive *Staphylococcus aureus* and Gram‐negative *Escherichia coli* [33].

Essential oil‐based plasma polymers, such as those from oregano, represent a novel class of biomaterials. Plasma polymerisation involves depositing thin, crosslinked polymeric films from volatile organic precursors (such as essential oils) in a reactive plasma environment. This process uses a plasma discharge (e.g., radio‐frequency plasma) to fragment and ionise organic molecules. Essential oils, such as oregano oil, are rich in bioactive compounds (e.g., terpenes and phenols) with functional groups suitable for polymerisation. These coatings retain or enhance the oils' inherent bioactivity, offering potential applications in medical devices, food packaging, and wound dressings. An oregano‐based plasma polymer (OPP), created through radio‐frequency plasma‐enhanced chemical vapour deposition (RF‐PECVD), has demonstrated biocompatibility with human dermal fibroblasts and antibacterial properties [34]. OPP promotes human dermal fibroblast growth, adhesion, and spreading. It also exhibits selective antibacterial and anti‐biofouling effects against *Pseudomonas aeruginosa* and *S. aureus* [34]. OPP retains chemical functionalities associated with oregano oil precursors within a stable plasma‐polymerised matrix, enabling broad‐surface applicability.

Despite prior reports demonstrating the antibacterial and biocompatible nature of oregano‐derived plasma polymers, their direct wound‐healing potential, particularly their influence on oxidative stress, immune modulation, and fibroblast‐driven repair, has not been explored. Therefore, in this study, we developed and characterised plasma‐polymerised coatings derived from oregano oil and assessed their suitability as multifunctional wound‐healing surfaces. Specifically, we performed comprehensive in vitro evaluations of antibacterial efficacy, cytocompatibility, and immune responses, together with antioxidant analysis to determine the ability of these coatings to mitigate oxidative stress. Most importantly, we conducted fibroblast migration and wound‐healing assays to assess the pro‐regenerative potential of these oregano‐based plasma polymer coatings. Through this integrated biological evaluation, we aimed to establish the translational potential of oregano‐derived plasma polymers as advanced, plant‐based biomaterials for active wound healing applications. Despite prior reports demonstrating the antibacterial and biocompatible nature of oregano‐derived plasma polymers, their direct wound‐healing potential, particularly their influence on oxidative stress, immune modulation, and fibroblast‐driven repair, has not been explored.

## 2 Materials and Methods

### 2.1 Deposition of Oregano‐Based Plasma Polymers

OPP coatings were deposited on 16 mm diameter glass coverslips (Proscitech, Kirwan, QLD) to enable the samples to fit into standard 24‐well plates for biological testing. Before deposition, all substrates were cleaned using a protocol of 5 min of sonication in isopropyl alcohol, rinsing, and 5 min of sonication in distilled water. The substrates were then allowed to air‐dry. The coating was performed using a radiofrequency plasma‐enhanced chemical vapour deposition (RF‐PECVD) reactor, the specifics of which have been described in prior literature [35]. A Florence flask, serving as the monomer reservoir for the plasma reactor, was filled with 200 μL of OEO (Sydney Essential Oil Company, Sydney, Australia, country of origin: Spain). According to the supplier's specifications, the OEO was obtained by steam distillation of the dried herb. Gas chromatography–mass spectrometry (GC–MS) analysis identified the principal constituents as carvacrol (~68%–70%), thymol (~8%–10%), p‐cymene (~7%–9%), and γ‐terpinene (~5%–6%), with minor components (< 2%) including β‐caryophyllene and linalool. The flow of the OEO into the reactor was carefully regulated using a vacuum stopcock, ensuring precise control over the monomer feed rate throughout the plasma polymerisation process. This setup enabled the consistent delivery of oregano oil vapour into the plasma chamber, thereby facilitating a uniform surface coating during the polymerisation reaction. The deposition of OPP was conducted under pulsed plasma conditions, utilising a 50% duty cycle and a frequency of 500 Hz, with a power setting of 50 W for 10 min.

### 2.2 Chemical Analysis Using X‐Ray Photoelectron Spectroscopy (XPS) and Fourier Transform Infrared Transmission (FTIR)

The chemical composition of OPP was characterised using x‐ray photoelectron spectroscopy (XPS). Survey spectra were acquired using a Kratos AXIS Ultra DLD spectrometer (Kratos Analytical Ltd., Manchester, UK) equipped with a magnetic charge compensation system and monochromatic AlKα radiation (hν = 1486.7 eV). The data were analysed using CasaXPS software (Casa Software Ltd., Teignmouth, UK). The C1s peak was recalibrated by assigning the carbon binding energies to 285 eV, and the deconvoluted peaks were fitted using the same software. FTIR spectroscopy (PerkinElmer Inc.) was employed to verify the preservation of oregano's functional groups in the OPP. The analysis was conducted using the attenuated total reflection (ATR) mode.

### 2.3 Contact Angle Analysis

The wettability of OPP was assessed by contact angle measurements using the sessile drop method with a KSV CAM 101 optical apparatus (KSV Instruments Ltd., Helsinki, Finland). A droplet of Milli‐Q water (8 μL) was deposited on the surface, and the contact angle was determined by referencing the droplet's baseline and the tangent of its boundary. Measurements were taken to account for the droplet's volume, height, and basal diameter.

### 2.4 Bacterial Cultures

*Staphylococcus aureus* ( *S. aureus* , ATCC 25923) and *Pseudomonas aeruginosa* (*P. aeruginosa*, PAO1, clinical isolate) were revived from −80°C glycerol stocks, plated on TSA, and incubated overnight at 37°C. These strains were selected as representative Gram‐positive and Gram‐negative pathogens commonly associated with medical device‐related infections and chronic wounds. Single colonies were cultured in 2 mL TSB for 18 h. OPP and control slides were UV‐sterilised (20 min) and placed in six‐well plates. Bacterial cultures were adjusted to OD_600_ = 1 (~10^9^ CFU/mL), diluted to ~10^6^ CFU/mL, and 2 mL was added to each well. The OPP and control samples were incubated at 37°C for 18 h.

### 2.5 Antibacterial Assessment

After incubation, the culture media were removed, and the wells were gently rinsed with 2 mL PBS for 1 min to eliminate planktonic cells. The LIVE/DEAD BacLight Bacterial Viability Kit (ThermoFisher Scientific, MA, USA) was used according to the manufacturer's protocol. Briefly, 1.5 μL/mL of Syto9 (excitation/emission 480/500 nm) and propidium iodide (excitation/emission 490/635 nm) were added to PBS. Samples were incubated in the dark for 15 min, then inverted onto a coverslip and imaged using an Olympus FV3000 confocal microscope (CLSM, Olympus, Tokyo, Japan). Three randomly selected ×40 confocal micrographs were acquired per sample under identical imaging settings for all groups, and live/dead surface‐associated bacteria were quantified using ImageJ (v1.53, NIH, WI, USA). Representative images shown in the figures were selected from datasets used for quantitative analysis.

### 2.6 Cell Culture

RAW 264.7 macrophage‐like cells (an Abelson leukaemia virus‐transformed cell line originating from BALB/c mice; ATCC TIB‐71, VA, USA) and human foreskin fibroblasts (HFF‐1, SCRC‐1041, ATCC, VA, USA) were cultured in Dulbecco's Modified Eagle's Medium (DMEM; ThermoFisher, CA, USA) enriched with 10% fetal bovine serum (FBS, Life Technologies, CA, USA) and 1% penicillin‐streptomycin (100 U/mL penicillin and 100 μg/mL streptomycin, Life Technologies, CA, USA). The cells were maintained at 37°C in a humidified atmosphere of 95% air and 5% CO_2_. For subculturing, cells were passaged when they reached approximately 70%–80% confluence using trypsin‐EDTA (Life Technologies, CA, USA). The medium was refreshed every 2–3 days.

### 2.7 MTT Cell Viability Assay

Macrophages are well‐suited for viability assays because they are critical in immune responses [36], making them a relevant model for studying cellular responses to biomaterials [37]. OPP‐coated and uncoated (control) coverslips (16 mm diameter) were placed in a 24‐well plate and sterilised under UV light for 20 min for this cell viability assay. RAW 264.7 cells were seeded at a density of 2 × 10^5^ cells per well and incubated for 48 h. Following incubation, cell viability was assessed for three samples of each plasma polymer and control coverslips using the MTT reagent (3‐(4,5‐Dimethylthiazol‐2‐yl)‐2,5‐diphenyltetrazolium bromide, Sigma‐Aldrich, MO, USA). The MTT assay, a colorimetric method, measures cellular metabolic activity to indicate cell viability and proliferation. It was chosen for its reliability and widespread use in cell viability testing. A stock solution of MTT was prepared at 0.5 mg/mL in phosphate‐buffered saline (PBS), pH 7.4. For each well containing 100 μL of medium, 10 μL of 5 mg/mL MTT was added. The plates were then incubated for 4 h at 37°C and 95% humidity in a 5% CO_2_ atmosphere. Afterwards, the solution was replaced with 200 μL of dimethyl sulfoxide (DMSO) and incubated in the dark at room temperature for 15 min. The absorbance was measured at 570 nm.

### 2.8 Cell Adhesion, Spreading, and Morphology

RAW macrophage‐like cells were seeded onto OPP‐coated and control coverslips and incubated for 48 h at 37°C, 95% humidity, and 5% CO_2_. After incubation, the cells were washed with phosphate‐buffered saline (PBS) and fixed with 4% paraformaldehyde (Sigma‐Aldrich, MO, USA) for 20 min at room temperature. The cells were then washed twice with PBS and permeabilised using 0.1% Triton X‐100 (Sigma‐Aldrich, MO, USA) in 1× PBS for 5 min. A blocking solution containing 1% bovine serum albumin (BSA, Sigma‐Aldrich, MO, USA) in PBS was applied for 30 min. Following two additional washes, the cells were stained with a 1:1000 dilution of TRITC‐conjugated phalloidin (Ex/Em 540/565, FAK100 kit, Sigma‐Aldrich, MO, USA) in PBS and incubated in the dark at room temperature for 60 min. Nuclei were counterstained by incubating the cells with 4′,6‐diamidino‐2‐phenylindole (DAPI, Sigma‐Aldrich, MI, USA; Ex/Em 359/461, FAK100 kit, Sigma‐Aldrich, MO, USA) in the dark for 5 min at room temperature. The cells were washed three times with PBS, and the coverslips were mounted onto microscope slides for imaging. Fluorescent images were captured using an Olympus FV3000 confocal laser scanning microscope (CLSM; Olympus, Tokyo, Japan). Representative images were selected from multiple randomly acquired fields, all collected under identical imaging settings, for all experimental groups.

### 2.9 Immune Response Evaluation

Macrophages play essential roles in wound healing, and our research suggests that oregano‐based plasma polymers could enhance these functions. This potential application of polymers in wound healing is a promising area for further exploration and development [38]. RAW 264.7 macrophage‐like cells were utilised to evaluate immune responses to OPP surfaces. To assess protein expression levels of the pro‐inflammatory markers IL‐6 and IL‐4, macrophages were seeded onto OPP‐coated and control coverslips and incubated for 48 h at 37°C, 95% humidity, and 5% CO_2_. After 48 h, the supernatant from each sample was collected and prepared for an enzyme‐linked immunosorbent assay (ELISA) to quantify IL‐6 and IL‐4. The ELISA was performed according to the manufacturer's instructions (ThermoFisher, MA, USA).

### 2.10 Antioxidant Evaluation of Oregano‐Based Plasma Polymers

RAW 264.7 macrophage‐like cells were cultured in Dulbecco's Modified Eagle's Medium (DMEM) supplemented with 10% fetal calf serum (ThermoFisher, MA, USA). The cells were seeded at a density of 2 × 10^5^ cells per well in a 24‐well plate and incubated overnight at 37°C, 5% CO_2_, and 95% humidity. One group of cells was treated with fresh media containing 100 ng/mL of bacterial lipopolysaccharide (LPS, Sigma‐Aldrich, MO, USA) to induce an inflammatory response and incubated overnight under the same conditions. After incubation, the DMEM media was replaced, and the cells were incubated with 10 μL of 2′,7′‐Dichlorofluorescein (DCF, Sigma‐Aldrich, MO, USA) in PBS for 30 min at 37°C, 95% humidity, and 5% CO_2_. The samples were then washed with PBS, and nuclei were stained with DAPI. Intracellular reactive oxygen species (ROS) were detected using an Olympus FV3000 confocal laser scanning microscope (CLSM; Olympus, Tokyo, Japan). ROS‐activated DCF was visualised using an excitation wavelength of 490 nm and an emission wavelength of 520 nm. Cellular fluorescence intensity was quantified using ImageJ version 1.54 J (NIH, MD, USA), accessed on 15 April 2024. Fluorescence intensity measurements were background‐subtracted and normalised to the corresponding control group prior to statistical analysis. Three randomly selected fields were analysed per sample under identical microscope acquisition settings for all groups. Cellular fluorescence intensity was quantified in ImageJ by grayscale conversion, ROI selection, threshold adjustment, intensity measurement, and background subtraction. The representative fluorescence micrographs shown were selected from datasets used for quantitative analysis.

### 2.11 In Vitro Scratch Assay

Our investigation into fibroblasts using wound scratch assays provides valuable insights into the cellular mechanisms underlying wound closure. By examining their migration and proliferation in response to injury, we can better understand the processes critical to wound healing and tissue regeneration [39]. Human foreskin fibroblasts (HFF‐1) were chosen as they are a standard model for studying dermal fibroblast migration and proliferation, key processes in wound repair involving extracellular matrix production and growth factor release [40]. The wound scratch assay was conducted in a 96‐well plate using an Incucyte SX5 Live‐Cell Analysis Instrument (Sartorius, Göttingen, Germany), housed within a cell incubator maintained at 37°C, 95% humidity, and 5% CO_2_. Round coverslips coated with OPP were affixed to the bottom of the 96‐well plate using Histoacryl (Braun, Melsungen, Germany). HFF‐1 cells, cultured in DMEM supplemented with 10% fetal bovine serum (FBS) and 1% penicillin–streptomycin, were seeded at a density of 1 × 10^5^ cells/mL and incubated for 24 h. Once the cells reached 80% confluence, uniform wound scratches were created using an Incucyte Wound Maker 96‐Tool. The wells were then washed with PBS to remove debris and dislodged cells, and fresh media was added. The 96‐well plate was placed in the Incucyte instrument, and images of wound closure were captured every 6 h over 30 h. Wound closure measurements were normalised to the initial wound area at 0 h for each condition using Incucyte image analysis software. Wound closure kinetics were calculated from the reduction in wound area and wound width over time. Representative images shown were selected from datasets used for quantitative analysis. Because cell proliferation was not experimentally inhibited, the measured wound closure reflects the combined contributions of fibroblast migration and proliferation.

### 2.12 Statistical Analysis

All statistical analyses and graphical representations were conducted using GraphPad Prism version 9.0.0 (121) (GraphPad Software, San Diego, CA, USA), accessed 18 October 2024. Each biological experiment was independently repeated three times (*n* = 3), and each biological replicate contained three technical replicates per condition to ensure reproducibility. For image‐based analyses, representative images were selected from datasets used for quantitative analysis. Quantification was performed using ImageJ from multiple randomly selected fields of view acquired under identical imaging settings for all experimental group. Data are expressed as mean ± standard deviation (SD). For the cytokine analysis of IL‐4 and IL‐6, ordinary one‐way analysis of variance (ANOVA) was performed, followed by Bonferroni post hoc testing to compare group means. To evaluate ROS intensity staining and wound‐healing assays, a 2‐way ANOVA was used, followed by Bonferroni post hoc tests for pairwise comparisons. Data normality was verified using the Shapiro–Wilk test prior to parametric analysis. Statistical significance was defined as *p* < 0.05.

## 3 Results and Discussion

### 3.1 Chemical Characterisation and Contact Angle of OPP

**FIGURE 1:**
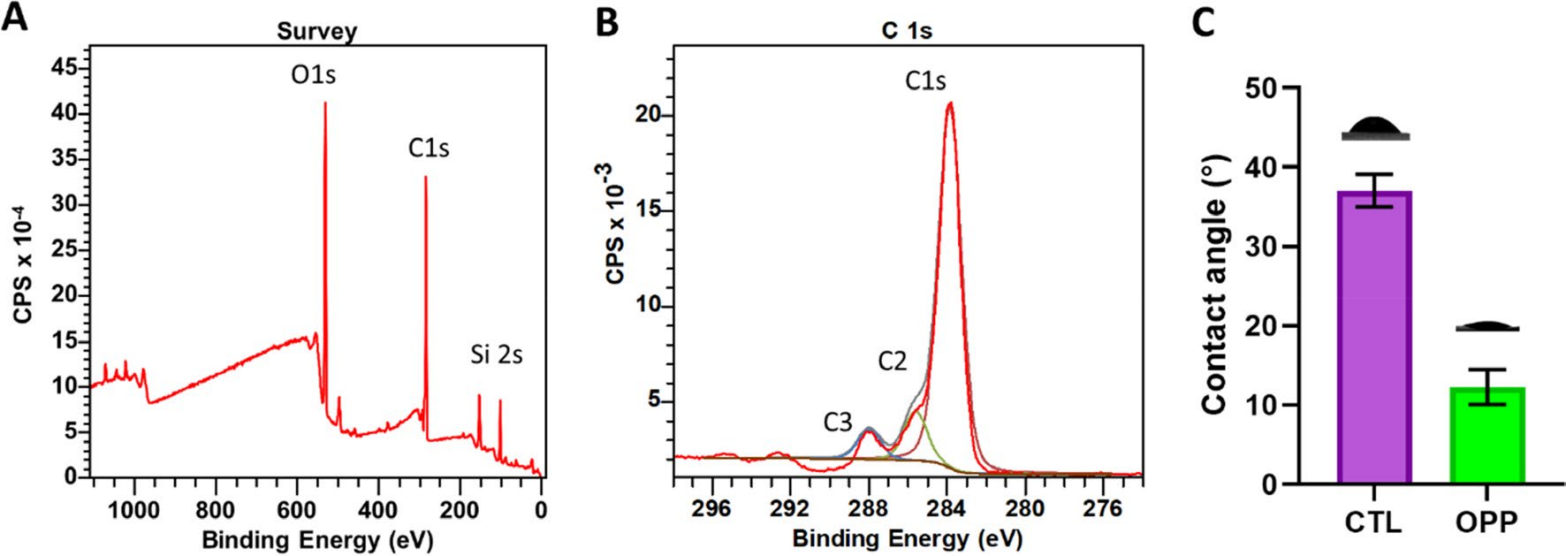
Chemical and physical characterisation of OPP. (A) Representative XPS survey, (B) high‐resolution C1s spectrum, and (C) water contact angle of OPP, as measured by the sessile drop technique. The data are plotted as mean ± SD (*n* = 3).

We performed detailed physicochemical analyses of the OPP coatings to better understand the surface properties responsible for the observed biological effects. Figure 1 presents the high‐resolution C1s spectra of OPP deposited using pulsed plasma (DC 50 W, 500 Hz, 10 min). The atomic percentages of the chemical elements comprising the OPP coatings are detailed in Table S1. The XPS survey spectrum in Figure 1A shows a prominent O1s peak at 532.63 eV, suggesting the presence of oxygen‐containing functional groups, including hydroxyl‐like species, consistent with the oxygenated chemistry of oregano oil precursors. These findings indicate partial retention of oxygenated functionalities during pulsed‐wave plasma deposition.

The C1s peak, depicted in Figure 1A, was deconvoluted into three components: a peak at 285 eV corresponding to aliphatic and aromatic carbons, a peak at 286.1 eV attributed to phenolic carbon atoms [38], and a component at 289 eV assigned to carboxyl (COOH) groups [41]. A COOH contribution can be attributed to the fragmentation and recombination processes occurring during plasma polymerisation. High‐energy plasma electrons break the precursor molecules into reactive species, and the extent of this fragmentation depends on the applied energy. These fragments then recombine in the gas phase and on the substrate surface, forming a highly crosslinked, irregular polymer structure distinct from conventional polymers [42]. These results are consistent with the FTIR data shown in Figure S1 and Table S2, as well as previous findings [34]. The high‐resolution spectra of O1s are presented in Figure S2. Although plasma polymerisation involves substantial precursor fragmentation and recombination, pulsed RF‐PECVD conditions may facilitate partial retention of oxygenated and phenolic‐like functionalities associated with oregano oil precursor. Consistent OPP formation and reproducible XPS and FTIR spectra suggest the stable incorporation of oxygenated and phenolic‐like functionalities, which may contribute to the observed antibacterial and antioxidant activity. However, XPS and FTIR analyses do not confirm the preservation of intact precursor molecules, and the plasma polymer is more appropriately described as a highly crosslinked coating that retains precursor‐derived chemical functionalities. Although the OPP coatings retained biological activity throughout the in vitro experiments performed under aqueous and serum‐containing culture conditions, comprehensive stability studies under wound‐relevant environments, including PBS, simulated wound fluid, protein‐rich media, and inflammatory conditions, remain necessary. Previous studies have demonstrated partial thickness reduction after prolonged aqueous immersion [34], underscoring the need for future investigations into the long‐term durability, chemical stability, and functional retention of coatings. The hydrophilicity of biomaterial surfaces is a crucial factor in tissue engineering, influencing the interaction of biological molecules and affecting cell adhesion, migration, proliferation, and survival [43]. Figure 1C presents the mean water contact angle (WCA) measured on the OPP surface compared to an untreated silicon substrate control (CTL). The WCA for the CTL group was 37° ± 2°. In contrast, the OPP measurement was 12° ± 2°, indicating its hydrophilic nature, consistent with its high hydroxyl group content, as confirmed by chemical characterisation. Increased hydrophilicity enhances protein adsorption, improving cell attachment and proliferation [43]. This creates a microenvironment with an adequate moisture content, a critical factor for rapid wound healing [44, 45]. These findings highlight the successful fabrication of hydrophilic OPP coatings that retain key functional groups, laying a strong foundation for their biological performance. Future studies should include additional plasma‐polymer controls, such as films deposited from inert or non‐bioactive organic precursors, to distinguish the specific effects of oregano‐derived chemistry from generic plasma surface modification, while also evaluating the stability and functional retention of OPP coatings in simulated wound fluid and under dynamic immersion conditions to confirm the persistence of their bioactive properties during prolonged clinical use.

### 3.2 Antibacterial Properties of OPP

**FIGURE 2:**
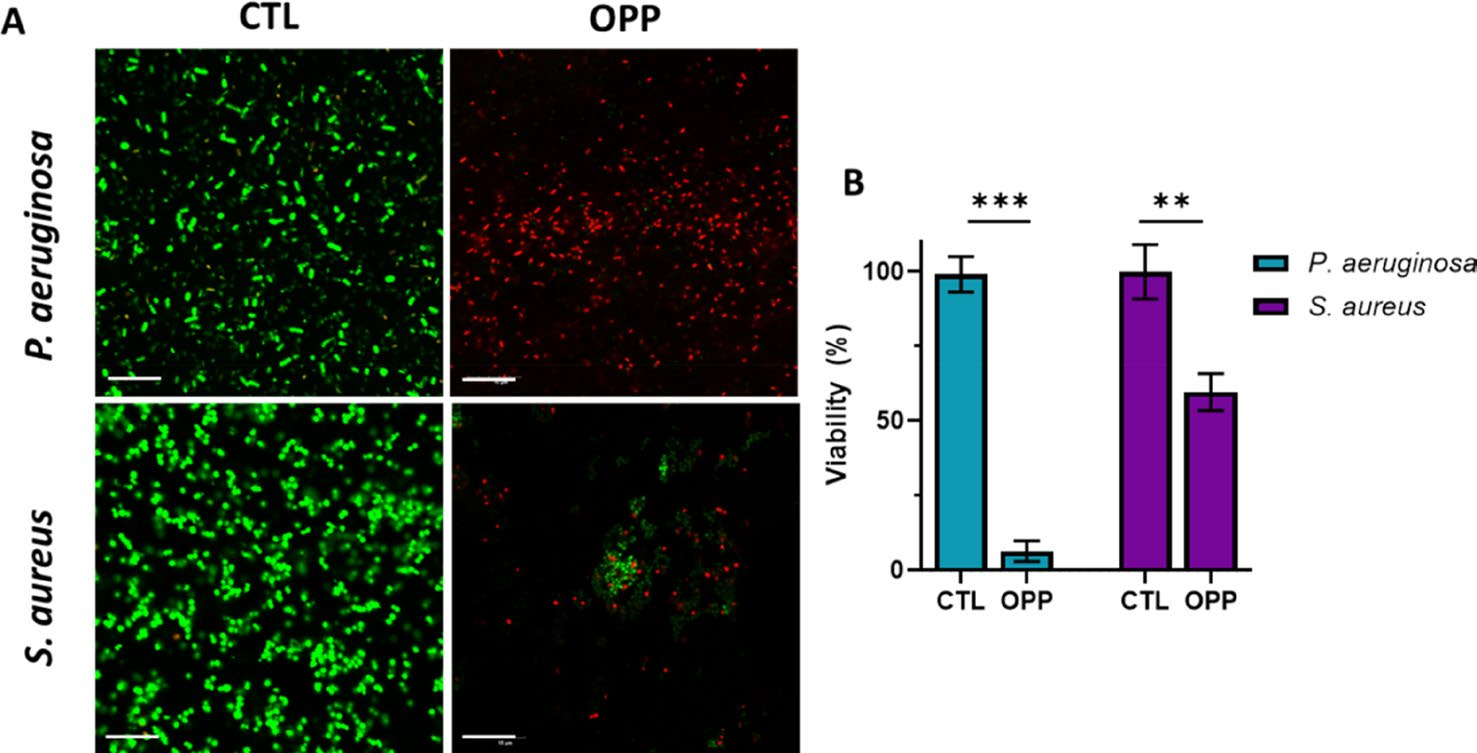
Viability of bacteria on OPP and control (CTL) glass slides. (A) Representative CSLM micrographs of *P. aeruginosa* (top row) and *S. aureus* (bottom row) stained with LIVE/DEAD Baclight Bacterial Viability Kit. Live cells appear green (Syto9), and dead cells appear red (propidium iodide). The scale bar represents 15 μm. (B) Quantification of live/dead cells using ImageJ. Data plotted as mean ± SD (*n* = 3), **p < 0.01 and ***p < 0.001. Representative images are shown from the datasets used for quantitative analysis.

Given the clinical relevance of infection control in wound healing, we next evaluated the antibacterial properties of the OPP coatings against representative Gram‐positive and Gram‐negative pathogens. CLSM revealed substantial differences in bacterial viability between control and OPP surfaces after 18 h of incubation. Both *P. aeruginosa* and *S. aureus* were abundant on uncoated slides, with green fluorescence predominating, indicating a high proportion of viable cells (Figure 2A). In contrast, OPP surfaces exhibited markedly reduced green fluorescence and increased red fluorescence, suggesting reduced bacterial viability and/or surface colonisation. Quantitative analysis of fluorescence images confirmed these observations (Figure 2B). Compared to controls, *P. aeruginosa* on OPP surfaces showed approximately 90% reduction in viable surface‐associated bacteria (*p* < 0.001), whereas *S. aureus* exhibited a more moderate reduction of approximately 40% relative to uncoated control surfaces (*p* < 0.01). The comparatively weaker effect observed against *S. aureus* may reflect species‐dependent differences in susceptibility, as Gram‐positive bacteria possess a thicker peptidoglycan cell wall that can reduce susceptibility to membrane‐disruptive phenolic functionalities compared with Gram‐negative bacteria such as *P. aeruginosa* [46, 47]. These findings are consistent with previous reports on the antimicrobial activity of plant‐derived compounds and demonstrate the utility of plasma polymerisation for the stable functionalisation of surfaces [48, 49]. Plasma‐polymerised coatings incorporating essential oils have been shown to significantly reduce bacterial viability, a critical challenge in chronic wound environments where persistent infections can delay healing. These findings highlight the potential of such coatings as surface‐active antimicrobial strategies, offering a promising approach for preventing or managing wound‐associated infections and promoting more effective healing.

Live/Dead staining combined with confocal imaging provides a semi‐quantitative assessment of surface‐associated bacterial viability; however, this method does not fully distinguish between bacterial killing, reduced adhesion, and bacterial detachment during washing procedures. Therefore, the observed reductions in bacterial coverage on OPP surfaces may reflect a combination of antibacterial and anti‐adhesive effects. Future studies will incorporate complementary quantitative approaches, including colony‐forming unit (CFU) enumeration, biomass quantification, and biofilm‐specific assays, to more comprehensively evaluate bacterial viability and surface colonisation.

### 3.3 Cytocompatibility, Adhesion and Spreading of the OPP

**FIGURE 3:**
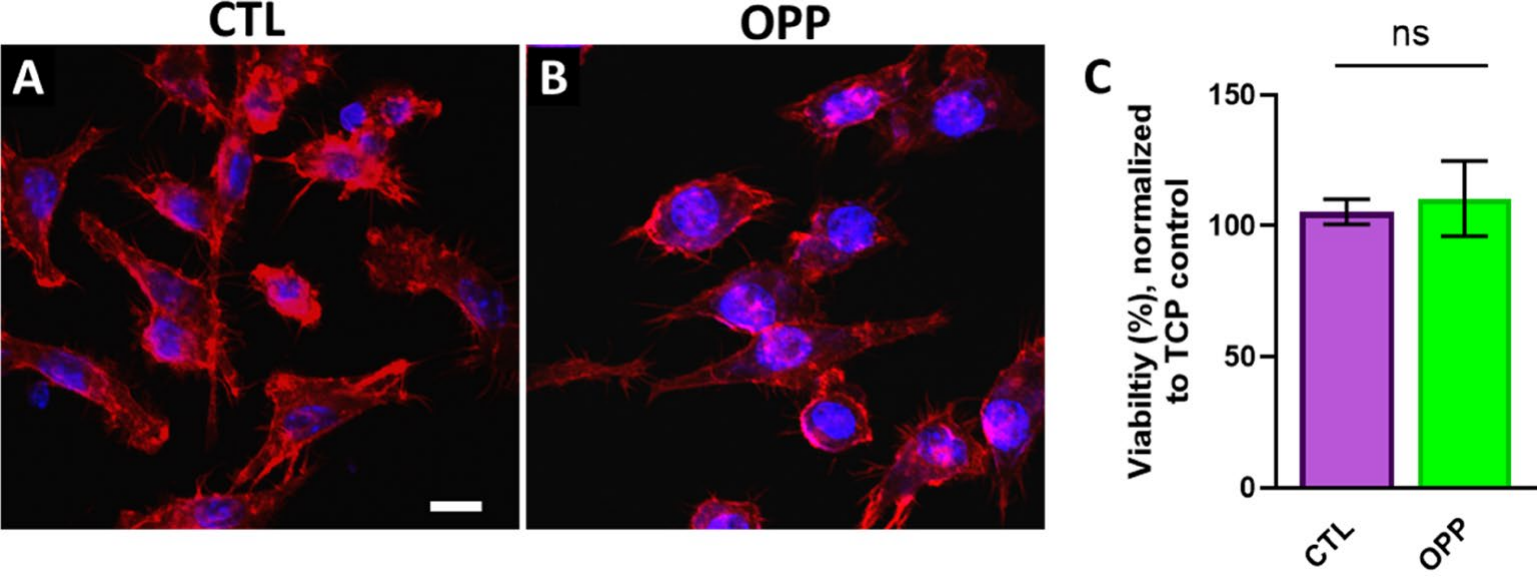
Fluorescent confocal microscopy images depict RAW 264.7 macrophages cultured on (A) untreated glass coverslip controls (CTL) and (B) OPP‐coated surfaces after 48 h of incubation. Cells were stained with phalloidin to visualise Actin filaments (red) and DAPI to label nuclei (blue). The scale bar corresponds to 10 μm. (C) Cell viability of RAW 264.7 macrophage‐like cells cultured on control and OPP surfaces after 48 h of incubation, as determined by MTT assay. Data plotted as mean ± SD (*n* = 3). Representative confocal images are shown from multiple randomly acquired fields.

Following the evaluation of antibacterial activity, it was crucial to determine whether the OPP coatings support cellular responses essential for tissue regeneration. To assess the biocompatibility of OPP coatings for potential wound healing applications, we next investigated their effects on cell viability, adhesion, and spreading. Figure 3A,B present fluorescence images illustrating the adhesion and spreading of RAW 264.7 macrophages on uncoated control coverslips and OPP‐coated surfaces. F‐actin filaments are visualised in red through staining with TRITC‐conjugated phalloidin, while nuclei are highlighted in blue using DAPI. F‐actin is essential for wound closure, supporting cell migration, adhesion, and proliferation [50]. Cells incubated on OPP surfaces displayed a healthy morphology comparable to that of cells on the control surface. Both surfaces exhibited well‐defined F‐actin filaments, indicative of focal adhesions and typical cytoskeletal organisation, supporting effective cellular attachment. The hydrophilicity of the OPP surface likely enhances anchorage‐dependent cell adhesion and proliferation, further confirming its ability to support cell integrity and functionality [34]. Cytocompatibility was assessed after 48 h by measuring cellular metabolic activity, an indicator of cell viability and proliferation, using the MTT assay. The macrophages incubated on OPP‐treated surfaces showed comparable values to the control, with 110.4% ± 8.2% and 105.3% ± 2.7%, respectively (*p* > 0.05) (Figure 3C). These findings demonstrate that OPP surfaces support macrophage adhesion, proliferation, and cytoskeletal organisation, highlighting their potential for wound healing and regenerative medicine.

### 3.4 IL‐4 and IL‐6 Cytokine Expression by OPP

**FIGURE 4:**
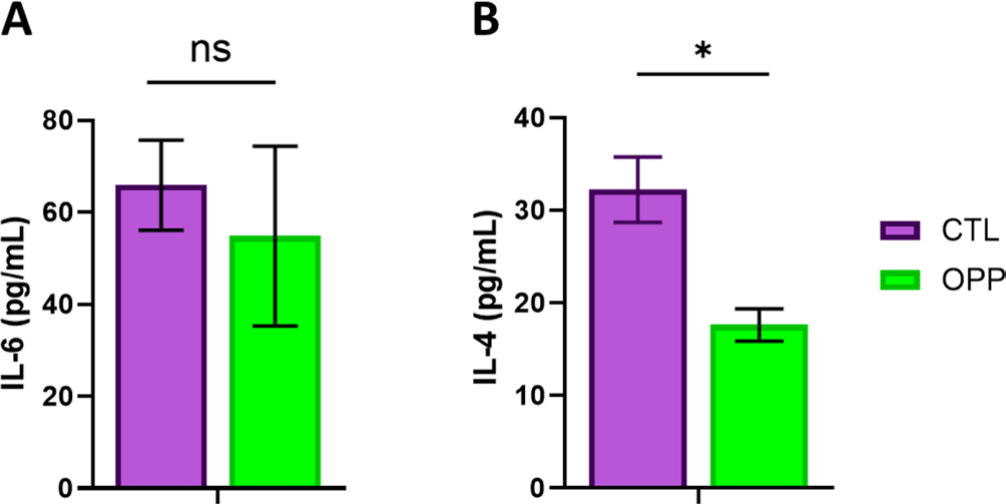
Preliminary cytokine expression analysis. Expression of IL‐6 (A) and IL‐4 (B) on RAW 264.7 macrophages incubated on untreated coverslips or OPP surfaces. Data plotted as mean ± SD (*n* = 3), *p < 0.05 and ns = nonsignificant.

To explore the immunomodulatory potential of OPP coatings, we analysed their impact on cytokine expression, focusing on IL‐4 and IL‐6 as key markers of anti‐inflammatory and pro‐inflammatory responses, respectively, in RAW 264.7 macrophage‐like cells. Increased secretion of IL‐4 and IL‐6 is commonly associated with macrophage activation and the initiation of inflammatory pathways [51]. It has been reported that oregano can potentially decrease IL‐6 production in LPS‐activated macrophages [52]. *Zataria multiflora*, a compound rich in carvacrol, has reduced IL‐4 levels in asthmatic animal models. Additionally, it decreased the expression of pro‐inflammatory cytokines, such as IL‐4, IL‐17, and TGF‐β, while increasing levels of anti‐inflammatory cytokines, including FOXP3 and IFN‐γ [53]. As illustrated in Figure 4A, the ELISA analysis revealed no statistically significant difference in IL‐6 expression on the OPP surface compared to the control. However, OPP significantly reduced IL‐4 expression compared to the control (17.6 ± 1.7 pg/mL vs. 32.2 ± 3.5 pg/mL; *p* < 0.05), as shown in Figure 4B. OPP did not induce elevated IL‐6 expression and reduced IL‐4 levels under the tested conditions, suggesting that the coatings influence macrophage cytokine responses without provoking a strong inflammatory reaction. This finding is particularly promising for wound‐healing applications, as previous studies have shown that direct application of oregano extracts can lead to irritation and inflammatory responses [25, 54, 55]. In contrast, OPP surfaces mitigate this pro‐inflammatory effect, potentially offering a gentler alternative for biomedical use. The ability of OPP to reduce the expression of key pro‐inflammatory cytokines without triggering irritation underscores its potential as a safe and effective material for wound healing. The observed modulation of IL‐4 and IL‐6 expression suggests that OPP coatings may influence macrophage behaviour; however, these findings alone are insufficient to define macrophage polarisation states or broader immunomodulatory outcomes. A more comprehensive assessment of macrophage polarisation, including markers such as TNF‐α, IL‐1β, IL‐10, CD86, CD206, Arg‐1, and iNOS, together with gene and protein expression analyses, will be required in future studies to fully characterise the immunomodulatory effects of OPP coatings.

### 3.5 OPP Surfaces Inhibit ROS Activation in RAW 264.7 Macrophages

**FIGURE 5:**
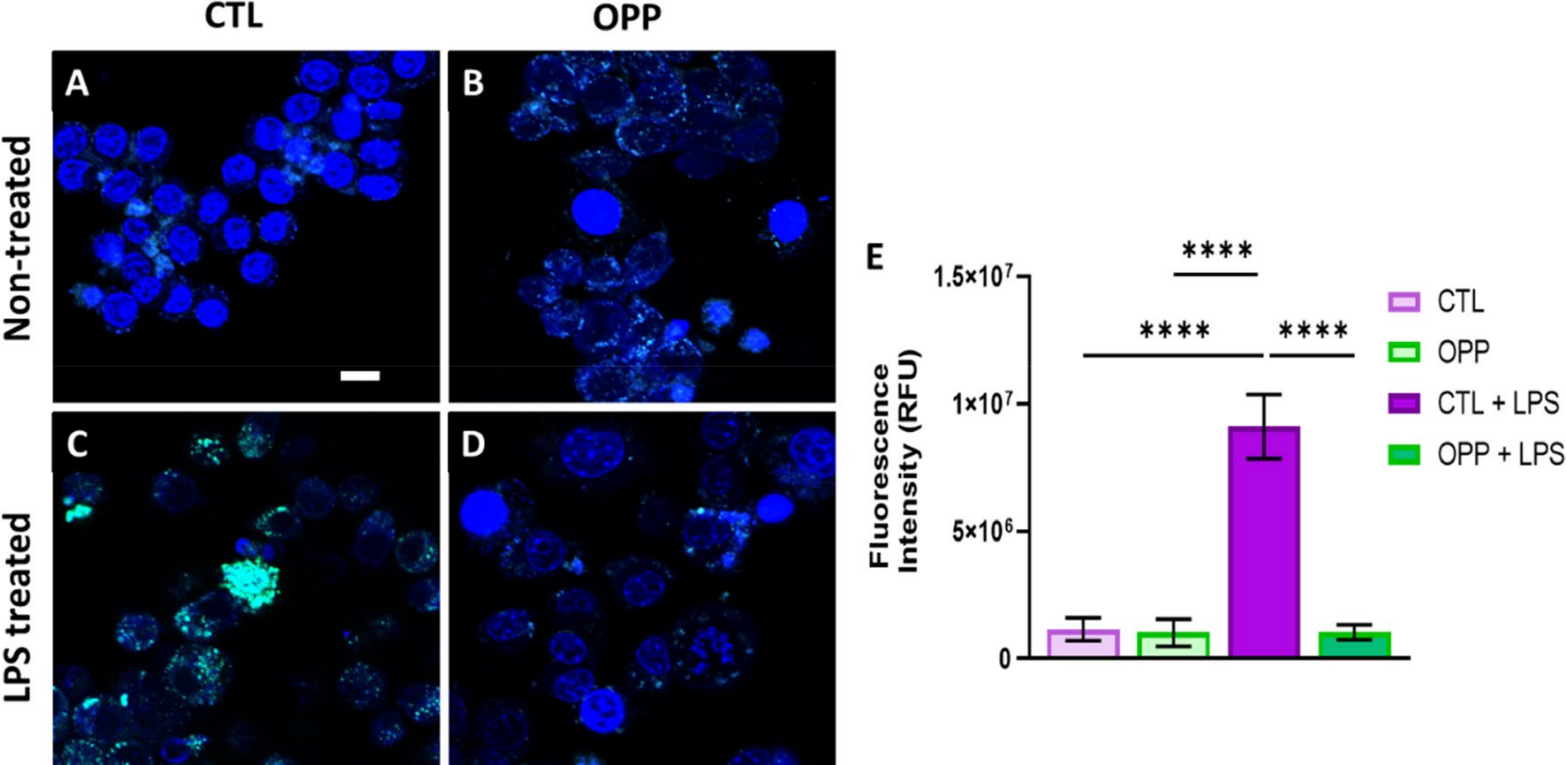
Quantification of the intracellular presence of ROS in RAW 264.7 macrophage‐like cells by fluorescence analysis. (A and B) Fluorescent micrographs of cells not stimulated by LPS are on either surface. (C and D) Fluorescent micrographs of cells stimulated by LPS on either surface. ROS levels were quantified by DCF fluorescence (Ex/Em 490/520 nm), in which oxidation of DCF by ROS results in measurable fluorescence. The scale bar represents 10 μm. (E) Normalised fluorescence intensities, quantified using ImageJ, are expressed as relative fluorescence units (RFU). The data are presented as mean ± SD (*n* = 3) and ****p < 0.0001. Representative fluorescence images are shown from datasets used for quantitative analysis.

To evaluate the antioxidative potential of OPP coatings, we investigated their ability to suppress ROS production in LPS‐stimulated RAW 264.7 macrophage‐like cells, as excessive ROS generation is a hallmark of chronic inflammation and impaired wound healing. Cellular ROS was measured using DCF dye as a fluorescent indicator [56]. Figure 5A,B indicate that the control and OPP samples exhibited no intracellular DCF fluorescence, indicating the absence of ROS activation. This suggests that OPP does not provoke oxidative stress or inflammatory responses in RAW 264.7 macrophages. Figure 5C,D demonstrate elevated intracellular ROS intensity in control samples treated with LPS, reflecting inflammation and oxidative stress. In contrast, the ROS intensity on LPS‐treated OPP surfaces remained low and comparable to that of the non‐LPS‐activated control, and was significantly lower than that in LPS‐activated RAW cells. The ROS intensity for the OPP + LPS samples was similar to the control (1.03 ± 0.29 × 10^6^ and 1.16 ± 0.45 × 10^6^, respectively; *p* > 0.05). However, the CTL + LPS samples exhibited a significantly higher ROS generation than the OPP + LPS samples (*p* < 0.0001) (Figure 5E). This finding indicates that OPP suppresses LPS‐induced ROS production in macrophages. As ROS signalling is closely associated with inflammatory pathways such as NF‐κB, it is possible that reduced oxidative stress may indirectly influence downstream inflammatory signalling [57], however, no direct pathway‐level analysis was performed in this study. Activation of NF‐κB is typically triggered by reactive oxygen species (ROS), leading to the transcription of inflammatory mediators contributing to macrophage activation and sustained inflammation. Given the established relationship between ROS and NF‐κB signalling reported in the literature [57], reduced ROS levels observed on OPP surfaces may potentially influence NF‐κB‐associated inflammatory responses, although this was not directly evaluated here. By preventing excessive NF‐κB activation, OPP may reduce the expression of pro‐inflammatory cytokines and iNOS, ultimately dampening the inflammatory response and lowering macrophage activation. This effect is particularly relevant in biomedical applications such as wound healing, where excessive inflammation can impair tissue regeneration. The ability of OPP to modulate this pathway suggests its potential as a promising material for reducing inflammation‐associated tissue damage while promoting a more controlled immune response. These results indicate that OPP surfaces can inhibit ROS production and macrophage activation, making them promising for controlling inflammation in wound healing. Although biofilm formation was not directly evaluated in this study, the combined antioxidant and antibacterial properties of OPP coatings suggest that these surfaces may also inhibit biofilm initiation and maturation, as oxidative stress is a key regulator of microbial adhesion and extracellular polymeric substance production [58]. Future studies will employ biofilm‐specific models, such as the CDC biofilm reactor and ex vivo wound models, to further verify these antibiofilm and surface‐protective effects under dynamic flow conditions. These findings demonstrate the potent antioxidant activity of OPP coatings, highlighting their ability to suppress ROS‐mediated macrophage activation and providing a promising strategy to mitigate inflammation and promote tissue regeneration in wound‐healing applications.

### 3.6 In Vitro Wound Healing Evaluation

**FIGURE 6:**
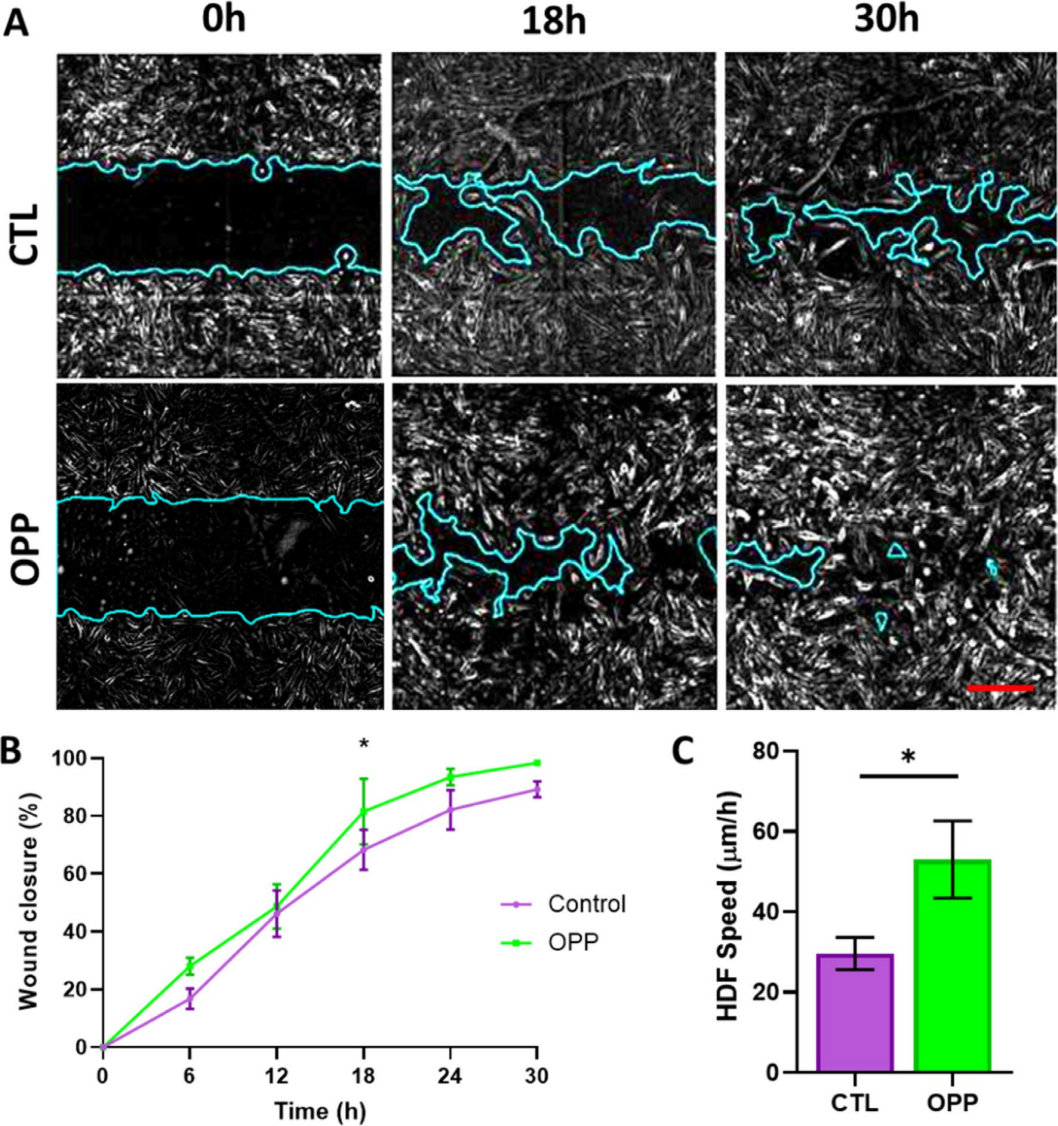
(A) Representative images of wound scratches on control (CTL) and OPP samples at 0, 18, and 30 h. The red scale bar in the bottom‐right image corresponds to 100 μm. (B) The wound closure percentage for both samples is plotted every 6 h, illustrating the progression of wound healing. (C) The wound closure rate is shown, with OPP surfaces demonstrating faster closure than the control. The data are plotted as mean ± SD (*n* = 3) and *p < 0.05. Representative images are shown from datasets used for quantitative analysis.

To determine the pro‐regenerative capacity of OPP coatings, we conducted an in vitro wound healing assay to assess their effects on fibroblast migration, a critical step in tissue repair and wound closure. An in vitro scratch assay was performed to evaluate the impact of OPP on cell migration and proliferation. HFF‐1 cells were selected for this experiment due to their pivotal role in wound healing and skin regeneration [39]. These cells are crucial for forming extracellular matrix components and for wound closure, making them an ideal model for studying cellular responses to treatment. The scratch assay enabled the evaluation of how OPP influences both the rate of cell migration across the wound gap and overall cell proliferation, providing insights into its potential as a wound‐healing promoter. Fibroblasts are activated during the inflammatory stage of wound healing and are attracted to the wound site. They also produce extracellular matrix, primarily collagen types I and III, which are essential for wound closure and growth factors necessary for new tissue formation [59]. Figure 6A displays 3 time points (0, 18, and 30 h). After creating a wound scratch on an HFF‐1 monolayer cultured on control and OPP surfaces, the wound closure percentage over time was plotted in Figure 6B. The results indicate that the wound closure rate was significantly faster on OPP surfaces than on the control, as illustrated in Figure 6C, suggesting enhanced healing potential on OPP surfaces. After 18 h, OPP achieved 81.5% ± 11% closure, while the control reached 68.2% ± 7% (*p* < 0.05). At 30 h, OPP samples reached 98.4% ± 0.7% closure, while the control reached 89.2% ± 2.7%. Although the difference was moderate, this early improvement in fibroblast migration may reflect the antioxidant and pro‐regenerative influence of oregano‐derived functional groups rather than a direct acceleration of wound closure.

The most significant difference in wound closure occurred within the first 18 h, with cell migration speeds of 29 ± 4 μm/h on CTL surfaces and nearly double that on OPP surfaces (53 ± 9 μm/h; *p* < 0.05), suggesting enhanced fibroblast‐mediated repair activity. This effect can be associated with the wound‐healing capacity of OEO, which comprises a plethora of molecules, including phenolic compounds, carvacrol, and thymol, as well as components like monoterpene hydrocarbons, such as p‐cymene and γ‐terpinene [60]. According to a recent study, topical applications of OEO may be a promising agent for wound healing. BALB/c mice infected with *Leishmania amazonensis* were treated with a 0.2% OEO‐based gel for 5 weeks [61]. This treatment led to a significant reduction in lesion size compared to the control groups, suggesting that OEO possesses therapeutic properties that can mitigate the effects of *Leishmania* infection. The gel's active compounds, particularly carvacrol and thymol, have been shown to exhibit wound‐healing properties in vivo, further supporting their potential as effective agents for promoting tissue regeneration and combating infection. These findings suggest that carvacrol and thymol may reduce the infection burden and promote lesion healing by modulating inflammatory responses and facilitating tissue repair. These results indicate that OPP coatings promote fibroblast‐mediated wound closure in vitro, highlighting their potential to support tissue regeneration and wound repair. However, because proliferation was not experimentally controlled, wound closure likely reflects combined contributions from both fibroblast migration and proliferation. Further studies incorporating proliferation‐inhibiting approaches, such as mitomycin‐C treatment, would help better isolate the specific contribution of fibroblast migration during scratch closure.

This study underscores OPP's potential to combat bacteria, enhance cell adhesion, suppress inflammation, and promote wound healing. The chemical analysis confirms the presence of hydrophilic functional groups, and in vitro assays demonstrate excellent cytocompatibility and decreased expression of pro‐inflammatory cytokines. OPP surfaces also inhibit ROS production in LPS‐treated macrophages and accelerate cell migration and wound closure in a scratch assay, positioning OPP as a strong candidate for wound‐healing applications. A recent study further validated our findings, demonstrating OEO's potent anti‐inflammatory and wound‐healing properties [62]. OEO reduced inflammation, DNA damage, and matrix metalloproteinase activity while enhancing cell motility in human keratinocytes, highlighting its potential to promote wound healing [62]. Another study showed that oregano essential oil (OEO) loaded into mucin‐based dissolving microneedles effectively treated hypertrophic scars in a rabbit model, further confirming OEO's in vivo efficacy [63].

While this study provides compelling in vitro evidence for the multifunctionality of OPP coatings, including antibacterial, antioxidant, and immunomodulatory effects, further investigations are needed to fully elucidate their clinical relevance. Notably, although our data suggest that OPP may attenuate macrophage activation, the underlying molecular mechanisms, such as modulation of the NF‐κB pathway, remain speculative without direct protein‐level analyses. Future work should include mechanistic validation using gene expression analysis, western blotting, or pathway‐specific reporter assays to confirm these regulatory effects. Moreover, in vivo studies (infected and diabetic animal models) will be crucial to evaluate long‐term biocompatibility, degradation kinetics, and therapeutic efficacy in a complex wound environment. Finally, integrating OPP with advanced wound dressing formats, such as microneedles, hydrogel scaffolds, or electrospun matrices, may enhance their therapeutic versatility. These future directions will help translate OPP from a promising bioactive coating into a clinically viable wound‐healing platform.

## 4 Conclusion

This study demonstrates the successful fabrication of OPP coatings via RF‐PECVD, thereby providing a multifunctional platform for advanced wound‐healing applications. The key novelty of this study lies in demonstrating, for the first time, that oregano‐derived plasma polymer coatings can function as multifunctional wound‐healing surfaces rather than solely antibacterial coatings. While previous studies established the antibacterial and cytocompatible nature of OPP coatings, the present work demonstrates, for the first time, their antioxidant activity, immunomodulatory effects on macrophages, suppression of ROS generation, and enhancement of fibroblast migration and wound closure. These findings significantly expand the therapeutic scope and translational relevance of oregano plasma polymers for regenerative medicine applications. OPP surfaces exhibited significant reductions in viable surface‐associated Gram‐negative and Gram‐positive bacteria, alongside excellent cytocompatibility, supporting macrophage viability, adhesion, and spreading. Additionally, they effectively modulate inflammatory responses by downregulating IL‐4 expression and suppressing ROS production in response to inflammatory stimuli. OPP coatings significantly accelerated fibroblast‐mediated wound closure in vitro, highlighting their pro‐regenerative and therapeutic potential. The retention of oxygenated and phenolic‐like functionalities derived from oregano oil precursors within a stable polymer matrix represents a novel approach to translating the benefits of plant‐based compounds into clinically relevant biomaterials. The novelty of this work lies in showing, for the first time, that OPP coatings can integrate antibacterial, antioxidant, immunomodulatory, and pro‐healing functions into a single, durable surface. Unlike conventional dressings that release bioactives, OPP forms a covalently bound, solvent‐free thin film that retains oregano's bioactivity while offering long‐term stability and scalability. While challenges such as precursor variability, plasma control, and sterilisation compatibility remain, the scalable RF‐PECVD process provides a viable route to large‐area biomedical coatings. Future work will include in vivo validation in infected and diabetic wounds, mechanistic studies of pathways such as NF‐κB, and detailed assessments of coating stability, degradation behaviour, and functional retention under simulated wound environments, including protein‐rich and inflammatory conditions. Clinically, these coatings address the multifactorial nature of chronic wounds by combating infection, oxidative stress, and inflammation, while promoting fibroblast‐driven repair. Their conformal nature enables use across diverse substrates, from dressings to implants, supporting strong translational potential.

## Funding

This work was supported by the National Health and Medical Research Council (GNT1194466) and Australian Research Council (DP180101254) for K.V. and Flinders Foundation for R.B.

## Ethics Statement

The authors have nothing to report.

## Conflicts of Interest

The authors declare no conflicts of interest.

## Supporting information


**Table S1:** The OPP's composition, eV position, and atomic percentage provide key information about its surface chemistry and elemental distribution. Analysis was performed in triplicate.
**Table S2:** FTIR spectra of functional groups of oregano‐based plasma polymers. These spectra provide detailed information about the polymer's chemical composition and surface functionalisation. FTIR analysis was performed in triplicate.
**Figure S1:** FTIR spectra of oregano essential oil and OPP reveal distinct chemical signatures for each substance. FTIR analysis was performed in triplicate.
**Figure S2:** High‐resolution XPS spectrum for O1s. FTIR analysis was performed in triplicate.

## Data Availability

The data that support the findings of this study are available from the corresponding author upon reasonable request.
