# Supplementary material for: Multifunctional Oregano‐Derived Plasma Polymer Coatings for Wound Healing Applications: An In Vitro Study
Source: Int Wound J. 2026 Jun 17;23(6):e70977. doi: 10.1111/iwj.70977 (PMC13275334; doi:10.1111/iwj.70977)
Supplement: Supplementary file 1 — Table S1: The OPP's composition, eV position, and atomic percentage provide key information about its surface chemistry and elemental distribution. Analysis was performed in triplicate. Table S2: FTIR spectra of functional groups of oregano‐based plasma polymers. These spectra provide detailed information about the polymer's chemical composition and surface functionalisation. FTIR analysis was performed in triplicate. Figure S1: FTIR spectra of oregano essential oil and OPP reveal distinct chemical signatures for each substance. FTIR analysis was performed in triplicate. Figure S2: High‐resolution XPS spectrum for O1s. FTIR analysis was performed in triplicate. [file IWJ-23-e70977-s001.docx]

Supporting Information

**Table S1.** The OPP's composition, eV position, and atomic percentage provide key information about its surface chemistry and elemental distribution. Analysis was performed in triplicate.

| **Name** | **Position** | **A %** |
| --- | --- | --- |
| O1s | 532.63 | 30.75 |
| C1s | 285.13 | 54.64 |
| N1s | 400.63 | 0.27 |
| Si 2p | 103.13 | 14.34 |

**Table S2.** FTIR spectra of functional groups of oregano-based plasma polymers. These spectra provide detailed information about the polymer's chemical composition and surface functionalization. FTIR analysis was performed in triplicate.

| **Wavelength (cm^-1^)** | **Functional group** | **Relationship** |
| --- | --- | --- |
| 2960-2866 | CH alkane strength  CH_2_ symmetric stretch | Carvacrol peak |
| 1700 | COOH Carboxyl bonds | Recombination of monomer molecules |
| 1457-1380 | Symmetric vibration CH_3_ |  |


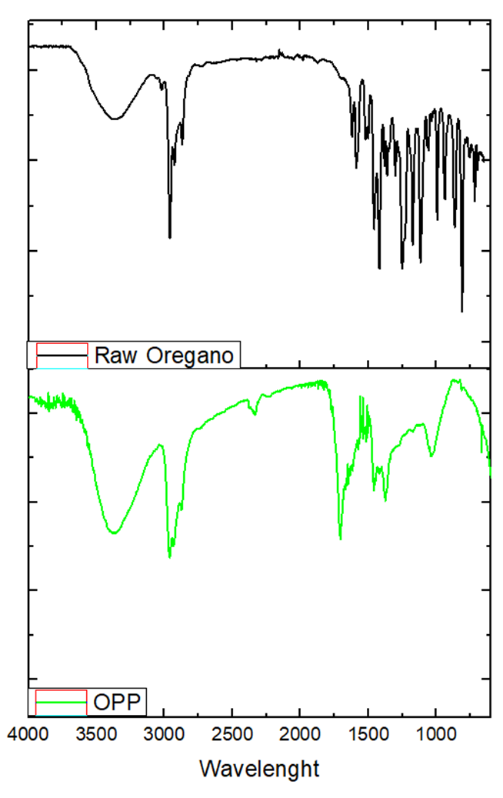


**Figure S1.** FTIR spectra of oregano essential oil and OPP reveal distinct chemical signatures for each substance. FTIR analysis was performed in triplicate.


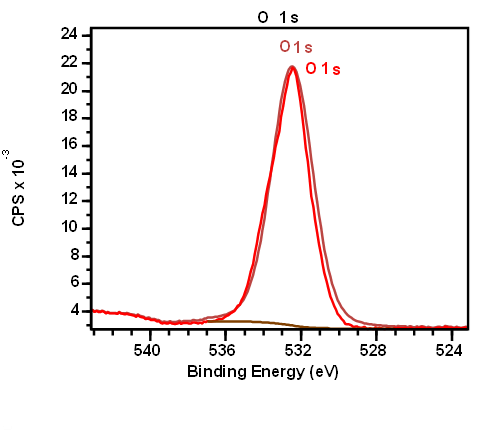


**Figure S2.** High-resolution XPS spectrum for O1s. FTIR analysis was performed in triplicate.
